# Interpretations of and management actions following electrocardiograms in symptomatic patients in primary care: a retrospective dossier study

**DOI:** 10.1007/s12471-019-01306-y

**Published:** 2019-07-12

**Authors:** L. M. E. Wagenvoort, R. T. A. Willemsen, K. T. S. Konings, H. E. J. H. Stoffers

**Affiliations:** Care and Public Health Research Institute (CAPHRI), Department of Family Medicine, Maastricht University, Maastricht, The Netherlands

**Keywords:** Electrocardiography, General practice, Quality of health care, Clinical skills, Diagnosis, Cross-sectional studies

## Abstract

**Background:**

The electrocardiogram (ECG) has become a popular tool in primary care. The clinical value of the ECG depends on the appropriateness of the indication and the interpretation skills of the general practitioner (GP).

**Objectives:**

To describe the use of electrocardiography in primary care and to assess the performance of GPs in interpreting ECGs and making subsequent management decisions.

**Methods:**

Three hundred ECGs, recorded during daily practice in symptomatic patients by 14 GPs who regularly perform electrocardiography, were selected. Corresponding data of the indications, interpretations and subsequent management actions were extracted from the associated medical records. A panel consisting of an expert GP and a cardiologist reviewed the ECGs and specified their agreement with the findings and actions of the study GPs.

**Results:**

The most common indications were suspicion of a rhythm abnormality (43.7%), ischaemic heart disease (42.7%) and patient reassurance (14.3%). The study GPs interpreted 53.3% of the ECGs as showing no (new or acute) abnormality, whereas supraventricular rhythm disorders (12.3%), conduction disorders (7.7%) and repolarisation disorders (7.0%) were the most frequently reported pathological findings. Overall, the expert panel disagreed with the interpretations of the study GPs in 16.2% of cases, and with the GPs’ management actions in 11.7%. Learning goals for GPs performing electrocardiography could be formulated for acute coronary syndrome, rhythm disorders, pulmonary embolism, reassurance, left ventricular hypertrophy and premature ventricular complexes.

**Conclusion:**

GPs who feel competent in electrocardiography performed well in the opinion of the expert panel. We formulated various learning objectives for GPs performing electrocardiography.

**Electronic supplementary material:**

The online version of this article (10.1007/s12471-019-01306-y) contains supplementary material, which is available to authorized users.

## What’s new?

- Common everyday indications to perform an electrocardiogram (ECG) in primary care are: suspicion of a rhythm abnormality, ischaemic heart disease and reassurance of the patient.
- Half of all ECGs recorded by general practitioners (GPs) revealed no (new or acute) abnormality. Frequent pathological findings were supraventricular rhythm disorders, conduction disorders and repolarisation disorders.
- Overall, GPs who feel competent in electrocardiography performed well in the opinion of the expert panel. However, the expert panel disagreed with 16.2% of the GPs’ ECG interpretations and 11.7% of the GPs’ management actions. The panel disagreed with both the interpretation and the subsequent management action in 5% of cases.
- Learning goals for GPs performing electrocardiography could be formulated for acute coronary syndrome, rhythm disorders, pulmonary embolism, reassurance, left ventricular hypertrophy and premature ventricular complexes.

## Introduction

### General background

The electrocardiogram (ECG) has become a frequently used and effective tool in primary care [1–3]. Nonetheless, the value of the ECG in primary care has been a recurrent topic of debate [1, 4–7]. The clinical value of the ECG strongly depends on the appropriateness of the indication and the competence of the physician interpreting the ECG [1, 2, 8–13]. Studies on the competence of general practitioners (GPs) have shown varying results [8, 13–16]. Competence is demanded since the quality of computer interpretation of ECGs is insufficient [17–20]. Besides, although Dutch primary care guidelines advocate the use of ECGs in specific situations (www.nhg.org/nhg-standaarden), clear guidelines on the use of ECGs in primary care settings are not available [21–23]. Currently, the actual performance regarding indications for and interpretation of the ECG among GPs is unknown.

To learn more about the use and usefulness of ECGs in primary care, we conducted a series of four studies (www.nhg.org/onderzoeken/het-ecg-de-nederlandse-huisartspraktijk-0). We expect these will support future ECG training for GPs. The first study addressed the competence of GPs in requesting and interpreting ECGs by means of a case vignette study [24]. In the remaining three studies, we have focussed on the performance of GPs in various real-life situations: ECGs recorded during out-of-office hours (to be published), ECGs performed during primary care cardiovascular risk management programmes (to be published) and ECGs carried out in symptomatic patients during day-care practice (this study).

### Objective

The objective of this study was to describe the use of everyday ECGs recorded for symptomatic patients in primary care and to assess the performance of GPs in interpreting ECGs and making subsequent management decisions.

## Methods

### Setting and design

Between September and October 2015, 14 GPs who regularly record and interpret ECGs themselves were recruited at an ECG training programme or by e‑mail for this retrospective dossier study. All ECGs that had been performed and interpreted by the GPs during office hours following a complaint reported to the GP or a finding observed during physical examination (‘symptomatic’) were analysed. An expert panel reviewed the interpretations of all ECGs and subsequent management actions.

### ECG data

**Fig. 1:**
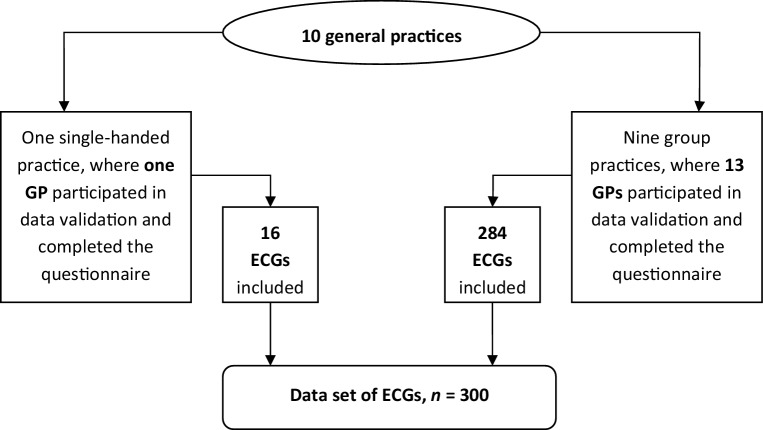
Participating practices, general practitioners (*GPs*) and collection of electrocardiograms (*ECGs*). We included ECGs that had been recorded and interpreted by the GPs during office hours in symptomatic patients, i.e. after a complaint reported to the GP or a finding observed during physical examination. Routinely performed ECGs for screening purposes were excluded

A master’s student of medicine (L.M.E. Wagenvoort) performed this study during a compulsory science elective of 18 weeks. In the practices of the participating GPs (Fig. 1), she reviewed the practice ECG archive and included ECGs performed during office hours in patients with certain symptoms or physical examination findings (‘symptomatic’). We excluded ECGs recorded in the context of cardiovascular risk management (‘screening ECGs’) or requested by other health care professionals. Patient characteristics and data on ECG indication, interpretation by the GP and subsequent management actions were traced in the electronic medical record system and copied to an anonymised case record form. Any uncertainties were clarified in a dialogue with the GP. Each GP also completed a short questionnaire on personal ECG skills (for an overview of all data, see Supplementary Table 1).

### Expert panel

Two highly experienced GPs and four cardiologists were included in the expert panel. Both expert GPs had worked as a cardiology resident for several years and successfully completed an acknowledged 2‑year training course on cardiovascular disease. For each ECG, one expert GP and one cardiologist reviewed interpretations and management actions. In the first round, the panel interpreted the ECG and accompanying data, blinded for the interpretation of the study GP. Then, the interpretation of the GP was revealed, and the experts indicated and explained the motivation for their (dis)agreement with the performance of the GP. If there was a difference in opinion between the two panel members, the second expert GP was consulted and determined the final panel verdict.

### Statistics

Descriptive analyses were made of GP characteristics, patient characteristics, ECG indications, interpretations and management actions, and the panel assessment using the Statistical Package for the Social Sciences (SPSS) version 21.

### Ethical considerations

This retrospective dossier study was not subject to the Dutch ‘Medical Research Involving Human Subjects Act’ (WMO). Case record forms did not contain personal data; all study data were anonymous. Any relevant disagreement of interpretation between the panel and the study GP was expected to become clinically irrelevant 3 months after the date of the visit. Therefore, we only included ECGs that had been recorded more than 3 months before the date of the actual data collection.

## Results

### Characteristics of GPs and ECGs

Details of the 14 participating GPs are shown in Supplementary Table 2. The median number of years of experience as a GP was 17 years. Four GPs had worked in a cardiology department in the past, 12 GPs had completed an ECG course. The reported median number of interpreted ECGs per month was 14.

Altogether, the ECGs of 300 individual patients were included (Fig. 1). The mean age of the patients was 61 years (range 14–92), 44.3% (133/300) were male, and in 82% (245/300) at least one cardiovascular risk factor or cardiovascular disease had been identified previously (see Supplementary Table 3 for more details).

### ECG indications

**Fig. 2:**
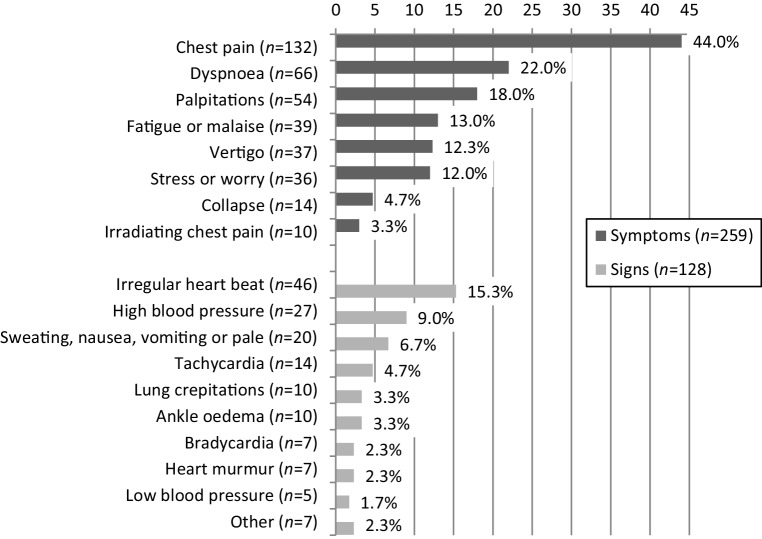
Percentage of presenting symptoms and signs in patients in whom the GP recorded an ECG, as reported in the medical records (*n* = 300). More than one symptom or sign may have been mentioned per ECG. ‘Other (*n* = 7)’ included: heart enlargement on chest radiography, biliary colic pain, panic attack, electrolyte abnormality, undefined, neurological deficit (2 ×) (*ECG* electrocardiogram, *GP* general practitioner)

**Fig. 3:**
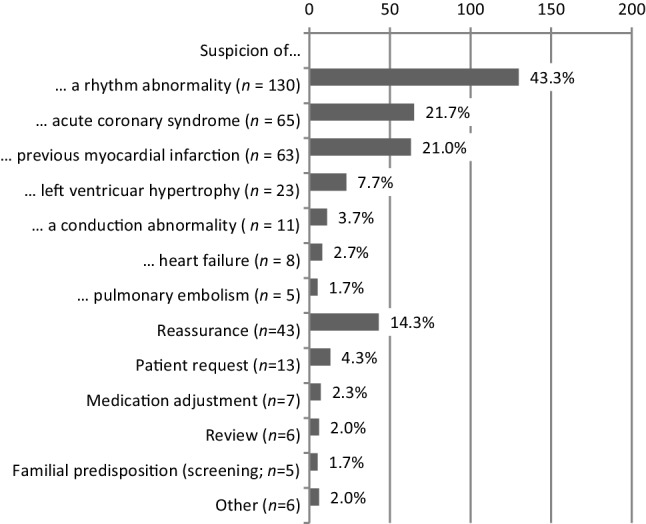
Indications for ECGs by GPs (percentage of all ECGs, *n* = 300). Since occasionally more than one indication was reported, the total number of reported indications (*n* = 385) exceeds the number of ECGs (*n* = 300). ‘Other (*n* = 6)’ included: myocarditis, pericarditis, hyperkalaemia, suspected long QT interval, aneurysm of abdominal aorta, no indication. All mentioned once (*ECG* electrocardiogram, *GP* general practitioner)

In Fig. 2, the signs and symptoms of patients in whom an ECG was performed are presented, and in Fig. 3 the indications for the ECGs are listed. The most frequently reported indications were (suspicion of) a rhythm abnormality (43.3%), an acute coronary syndrome (21.7%) or an unknown old myocardial infarction (21.0%). Patient ‘reassurance’ (14.3%) was another commonly reported indication.

### ECG interpretations

All ECG interpretations as documented by the GPs are listed in Tab. 1. The GPs interpreted 54.3% (163/300) of the ECGs as showing ‘no (new or acute) abnormality’. Supraventricular arrhythmia was reported in 36 (12.0%) cases. In 28 (9.3%) cases no interpretation could be extracted from the patient record, and in 10 (3.3%) cases the GP had requested the cardiologist to interpret the ECG.

**Table 1:** Frequencies of ECG interpretations reported by the GP

GP’s ECG interpretation	*n*	Percentage of all ECGs (*n* = 300)
No (acute or new) abnormalities	163	54.3
– Normal	121	40.3
– No changes compared to previous ECG	19	6.3
– No acute pathology	23	7.7
Sinus node arrhythmia	17	5.7
– Sinus arrhythmia	8	2.7
– Sinus tachycardia	5	1.7
– Sinus bradycardia	4	1.3
Supraventricular arrhythmia	36	12.0
– Atrial fibrillation	30	10.0
– Atrial flutter	4	1.3
– Ectopic atrial rhythm	1	0.3
– Premature supraventricular complex	1	0.3
Premature ventricular complex	14	4.7
Conduction abnormality	28	9.3
– First-degree AV block	4	1.3
– Second-degree AV block	1	0.3
– Ventricular pre-excitation (Wolff-Parkinson-White pattern)	1	0.3
– Nodal rhythm	2	0.7
– Right bundle branch block	10	3.3
– Left bundle branch block	6	2.0
– Left anterior fascicular block	3	1.0
– Trifascicular block	1	0.3
QRS axis deviation	14	4.7
– Left axis deviation	13	4.3
– Right axis deviation	1	0.3
Repolarisation abnormalities	21	7.0
– Non-specific ST/T abnormality	8	2.7
– ST/T abnormality suggestive of acute ischaemia	13	4.3
Abnormalities suggestive of old myocardial infarction	18	6.0
– Non-acute signs of myocardial ischaemia	9	3.0
– Slow R progression	3	1.0
– Pathological Q wave(s)	6	2.0
Left ventricular hypertrophy	6	2.0
Abnormal, not specified	3	1.0
ECG interpretation of the study GP missing	38	12.7
– Missing	28	9.3
– ECG interpreted by cardiologist	10	3.3

Since more than one ECG interpretation per ECG was reported in several cases, the total number of interpretations (*n* = 358) exceeds the number of ECGs (*n* = 300)

*ECG* electrocardiogram*, GP* general practitioner*, AV* atrioventricular

### Management actions following the ECG

Management actions by the GP following the ECG are listed in Tab. 2. In 130 of 300 (43.3%) cases no further action was taken. In most cases, actions following the ECG were taken by the GP alone (187/300, 62.3%). In 113 of 300 cases (37.7%) a specialist, mainly a cardiologist, was involved. Among these were 42 cases of immediate referral to a cardiologist, 30 routine referrals and 29 cases of consultation by telephone/telefax (of whom two patients were referred subsequently).

**Table 2:** Frequencies of management actions taken by the GP after the ECG (*n* = 300)

No specialist involved (*n* = 187)	No action	130 (43.3%)
	Further diagnostic evaluation by GP	39 (13%)
	Medication adjustment by GP	13 (4.3%)
	Medication and further diagnostic evaluation by GP	5 (1.7%)
Specialist involved (*n* = 113)	Further diagnostic evaluation and routine referral to cardiologist	2 (0.7%)
	Medication and routine referral to cardiologist	1 (0.3%)
	Routine referral to cardiologist	27 (9%)
	Telephone consultation with cardiologist (followed by medication adjustment 6, further diagnostic examination in primary care 4, both medication adjustment and further examination 1, referral 2)	29 (9.7%)
	Immediate referral to cardiologist	42 (14%)
	Referral to other specialist	12 (4%)

*GP* general practitioner, *ECG* electrocardiogram

When the GP interpreted the ECGs as showing ‘no (new) abnormality’ (*n* = 160), no further management action was taken in 63% (100/160) of cases, and further diagnostic evaluation was planned in 14% (23/160). In 43 ECG cases for which the indication was ‘reassurance’, 86% of the ECGs were followed by ‘no action’. When the indication was ‘suspicion of acute coronary syndrome’, the GP referred 42% of the patients (27/65) immediately in. In nine of these 27 referred cases, the ECG showed no abnormal findings. For the most frequently reported symptom preceding the ECG, namely ‘chest pain’ (*n* = 132), the most frequent subsequent actions were ‘no action’ in 53 cases (40%), and ‘immediate referral’ in 29 cases (22%), including nine cases in which the ECG revealed no abnormalities.

### Comparison to expert panel

**Fig. 4:**
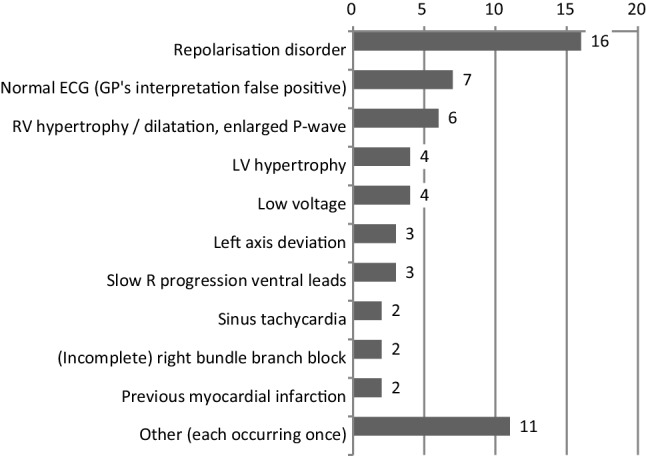
Absolute numbers of ECG abnormalities missed or incorrectly interpreted by GPs. In 43 out of 300 ECGs, 60 missed or incorrectly interpreted ECG abnormalities were described by the expert panel. ‘Other (*n* = 11)’ included: sinus bradycardia, supraventricular premature complex, atrial flutter, non-sustained ventricular tachycardia, 1st degree AV block, 3rd degree AV block, right axis deviation, horizontal axis, ‘pathological’ Q‑wave, S1Q3 pattern, lead reversal. All mentioned once (*AV* atrioventricular, *ECG* electrocardiogram, *GP* general practitioner, *LV* left ventricular, *RV* right ventricular)

Due to missing GP interpretations (*n* = 28) or poor ECG quality (*n* = 7), the panel could not review the GP’s interpretation of 35 of the 300 ECGs (12%). There was full agreement (ECG interpretation plus management) between the expert panel and GPs on 207 of the 300 (69%) cases. In 43 of the 265 (16.2%) assessable ECG interpretations, the panel disagreed on one or more aspects of the ECG interpretation of the study GP (Fig. 4). The panel disagreed with the GP’s management action in 35 of the 300 (11.7%) cases; in 15 of these cases (5% of all ECGs) the panel disagreed on the interpretation as well as the management.

In most cases in which they disagreed with the GP’s management, the panel advised further examination, medication adjustment or referral to the outpatient cardiology clinic. However, in two cases, contrary to the GP’s original decision, the panel advised making an immediate referral: one case of suspected acute coronary syndrome where the panel agreed that the ECG revealed no relevant abnormalities, and one case of bradycardia that had been missed by the GP.

## Discussion

### Main findings

In this study of 300 ECGs recorded during daily practice in symptomatic patients by 14 GPs, the most frequently reported indications were (suspicion of) a rhythm abnormality, ischaemic heart disease and patient ‘reassurance’. In 86% of the latter ECG cases, the ECG was not followed by any action. The GPs interpreted half of the ECGs as revealing no (new or acute) abnormality. Supraventricular rhythm disorders, conduction disorders and repolarisation disorders were the most frequently reported pathological findings.

In around 60% of the analysed cases, the ECG was followed by either no further action, a medication change or an additional diagnostic test. In almost 40% of cases, a cardiologist was consulted immediately or at a later time. In 27 of 65 cases of ‘suspected acute coronary syndrome’, the GP referred immediately, including 9 cases without an ECG abnormality.

The panel disagreed with the GPs’ interpretations in 16.2% of the assessable ECGs. The panel disagreed with the GPs’ management actions in 11.7% of all ECG cases. In most of the latter cases, the panel would have advised further examination, medication adjustment or referral to the outpatient cardiology clinic. However, in two cases, contrary to the GP’s original decision, the panel would have advised referring immediately: one case of suspected acute coronary syndrome with a normal ECG and one case of (missed) bradycardia.

### ECGs performed in symptomatic patients by GPs during office hours

In 84% of the assessable ECGs, the panel agreed with the interpretation of the study GP. This result is similar to those of other studies, in which agreement between the GPs and a cardiologist was found in 80–90% of cases [4, 13]. The panel entirely disagreed with (both the interpretation and the subsequent management action of) the study GP in 5% of cases. However, management actions may have been postponed in some cases. The rather low percentage of disagreement on interpretation and management may be regarded as an indication that GPs master electrocardiography in everyday symptomatic cases satisfactorily.

However, from the points of disagreement between GP and expert panel, learning goals for GPs performing electrocardiography could be formulated for acute coronary syndrome, rhythm disorders, pulmonary embolism, reassurance, left ventricular hypertrophy and premature ventricular complexes (Tab. 3). In addition, our previous vignette study on the competence of GPs in requesting and interpreting ECGs revealed that GPs showed poor diagnostic accuracy for left anterior fascicular block and incomplete right bundle branch block [24].

**Table 3:** Learning goals derived from the expert panel’s observations in this study to improve GPs’ competence in interpreting ECGs recorded in primary care patients

Observation	Learning goal
Several patients with chest pain were referred immediately although the electrocardiogram (ECG) was normal. This is in accordance with guidelines stating that an ECG is not suitable to rule out acute coronary syndrome (ACS) in acute situations [1]. However, several chest pain patients with normal ECG findings were not referred immediately	Although the causal relationship between the normal findings on the ECG and the subsequent non-referral is difficult to establish, it seems reasonable to conclude that when teaching interpretation of ECGs to general practitioners (GPs), one learning goal should be that ECGs are unsuitable to rule out ACS in acute chest pain cases
In one case, the expert panel disagreed on the GP’s exclusion of a rhythm disorder based on a negative ECG, which was recorded at a time when the patient was not experiencing the reported complaints	Especially for confirming or excluding rhythm disorders, an ECG should be recorded when the symptoms are being experienced
In one ECG, the indication was ‘suspicion of pulmonary embolism’. Since the study GP interpreted this ECG as ‘normal’ and no management action followed, it appeared that the study GP used the ECG to exclude pulmonary embolism	The negative predictive value of such an ECG is too low, leading to the conclusion that exclusion of pulmonary embolism is not possible based on an ECG
A normal diagnostic test does not necessary reassure patients [25]. Therefore, using an ECG for reassurance can be regarded as doubtful. However, the expert panel considered reassurance to be an important part of a GP’s work, leading to a high level of agreement on ECGs performed to reassure patients	Thus, reassurance seems feasible. However as pointed out earlier, the negative predictive value of an ECG in ruling out rhythm disorders in the absence of symptoms, or ACS, is low
The expert panel considered the indication ‘left ventricular hypertrophy’ (LVH) often to be unfounded, since hypertension should be treated properly irrespective of the presence of LVH	The indication ‘left ventricular hypertrophy’ is doubtful
In one ECG, the GP interpreted the series of broad QRS complexes as multiple premature ventricular complexes (PVCs), whereas the expert panel described this ECG as non-sustained ventricular tachycardia	Although PVCs are usually innocent in primary care, three or more PVCs in a row, as well as fusiform or multiform PVCs, should be viewed with caution. Referral to a cardiologist for further risk assessment of ventricular rhythm disorders is necessary

The precise role of ECGs in the clinical reasoning of the GP, the potential of eHealth collaboration between GP and cardiologist, and possibly machine learning in interpreting ECGs outside the cardiology practice may be topics for future research.

### Strengths and limitations

To our knowledge, our study is the first to provide insight into indications for, interpretations of, and management actions following ECGs recorded in everyday symptomatic patients in primary care. We analysed a rather large sample of 300 real-life ECGs performed and interpreted by GPs who do this routinely. Therefore, we regard our results to be representative for ECGs recorded by GPs who feel competent in electrocardiography. Since we analysed historical data, the behaviour of the participating GPs was not influenced by the study. We collected as much clinical information as possible to enable the expert panel to assess each ECG in its clinical context. Our panel assessment was based on the independent judgement of at least two panel members, a cardiologist and a GP. The panel disagreed with the management action only if there were clear reasons for this disagreement.

Some limitations need to be acknowledged. We described the use of electrocardiography in primary care and had a panel judge the ECGs that were performed as they were. However, we were not able to judge the usefulness of electrocardiography in primary care, since the decisions to perform electrocardiography had already been made and correlations with management decisions were not assessable due to the retrospective design of the study. Our results can only be generalised to GPs who feel sufficiently competent in interpreting ECGs. The quality of the extracted data depended on the documentation in the medical records, although we clarified uncertainties in a conversation with the GP. To assess whether abnormalities were new and thus more relevant was only possible in a minority of cases where previous ECGs were available. The members of the panel judged the cases only on paper, and may have missed factors that were decisive for the participating GPs, such as the complete clinical picture, including background knowledge of the patient. Furthermore, the panel had no access to previous ECGs. Moreover, due to the pragmatic study design, the panel was unable to determine the precise connection between the study GP’s ECG interpretation and the subsequent management action. In this light, the (dis)agreement of the panel with the GPs’ decisions is to a certain extent arbitrary. Several patient factors can contribute to a management action that seems erroneous when assessing the case on paper, but may be defendable in a real-life context. Finally, since we did not have any follow-up data on the patients, we do not know whether the disagreements on management actions were actually indicative of adverse clinical outcomes.

## Conclusions

This study indicates that GPs who feel competent in electrocardiography perform satisfactorily with regard to both the interpretation of ECGs recorded in symptomatic patients and to subsequent management actions. To further improve the ability of GPs to interpret ECGs, we have formulated various learning objectives for GPs performing electrocardiography.

## Caption Electronic Supplementary Material

**Supplementary Table 1 **Overview of registered study data

**Supplementary Table 2 **Characteristics of the general practitioners (*N* = 14)

**Supplementary Table 3 **Characteristics of the patients (*N* = 300)
