# Supplementary material for: Interpretations of and management actions following electrocardiograms in symptomatic patients in primary care: a retrospective dossier study
Source: Neth Heart J. 2019 Jul 12;27(10):498–505. doi: 10.1007/s12471-019-01306-y (PMC6773798; doi:10.1007/s12471-019-01306-y)
Supplement: Supplementary file 1 — Supplementary Table 1 Overview of registered study data [file 12471_2019_1306_MOESM1_ESM.docx]

**Supplementary Table 1** Overview of registered study data

| **Main category** | **Categorisation / variables measured** |
| --- | --- |
| *Information about GP’s ECG skills* | - years of service - estimated number of ECG interpretations per month - the use of the computer interpretation - received education in ECG interpretation |
| *Patient characteristics* | - sex - age - data on medical history - cardiovascular risk factors - cardiovascular morbidity |
| *Indications for ECG* | - suspicion of a rhythm abnormality - suspicion of unknown old myocardial ischemia - suspicion of acute coronary syndrome - suspicion of heart failure - suspicion of left ventricular hypertrophy - medication adjustment - reassurance - patient request - screening because of familial predisposition - suspicion of conduction abnormality - suspicion of pulmonary embolism - review |
| *Interpretation of ECG by GP (23)* | - no (new or acute) abnormalities - sinus node arrhythmia - supraventricular arrhythmia - ventricular extra-systole - conduction abnormality - QRS axis deviation - repolarization abnormalities - abnormalities suggestive of old myocardial infarction - left ventricular hypertrophy - abnormal, not specified - ECG interpretation of study GP missing |
| *Management actions following ECG* | - no action - cardiovascular medication adjustment - further diagnostic examination - routine referral to a cardiologist - direct referral to a cardiologist (within one day) - telephonic consultation with the cardiologist - referral to specialist (other than cardiology) |
| *Expert panel assessment* | - blinded interpretation of the ECG - agreement with the study GP’s interpretation - expert’s choice of management action (expert GP only) - agreement with study GP’s management action (expert GP only) - highlighting a missed relevant ECG abnormality |

*Abbreviations: ECG electrocardiogram, GP general practitioner*
