# Supplementary material for: Interpretations of and management actions following electrocardiograms in symptomatic patients in primary care: a retrospective dossier study
Source: Neth Heart J. 2019 Jul 12;27(10):498–505. doi: 10.1007/s12471-019-01306-y (PMC6773798; doi:10.1007/s12471-019-01306-y)
Supplement: Supplementary file 2 — Supplementary Table 2 Characteristics of the general practitioners (N = 14) [file 12471_2019_1306_MOESM2_ESM.docx]

**Supplementary Table 2** Characteristics of the general practitioners (N=14)

| Number of males (%) | 8 (57%) |
| --- | --- |
| Years of service - mean [range] | 17 [5-30] |
| Self-reported number of ECG interpretations per month - mean [range] | 14 [2-50] |
| Number of GPs who report never to consider the automatically generated computer interpretation (%) | 3 (21%) |
| Number of GPs who had participated in an ECG training program (%) | 12 (86%) |
| Number of GPs who had been a cardiology resident (%) | 4 (29%) |

*Abbreviations: ECG electrocardiogram, GP general practitioner*
