# Supplementary material for: Interpretations of and management actions following electrocardiograms in symptomatic patients in primary care: a retrospective dossier study
Source: Neth Heart J. 2019 Jul 12;27(10):498–505. doi: 10.1007/s12471-019-01306-y (PMC6773798; doi:10.1007/s12471-019-01306-y)
Supplement: Supplementary file 3 — Supplementary Table 3 Characteristics of the patients (N = 300) [file 12471_2019_1306_MOESM3_ESM.docx]

**Supplementary Table 3** Characteristics of the patients (N=300)

| Age (years) | Median 64,  Mean 61 (range 14-92) |
| --- | --- |
| Number of males (%), females (%) | 133 (44.3%), 167 (55.7%) |
| Cardiovascular risk factor (more than one risk factor or disease per patient possible)  None (%)  Hypertension (%)  Smoking (%)  Positive family history (%)  Hypercholesterolemia (%)  Diabetes Mellitus (%)  Chronic kidney disease (%)  Reumatoid Artritis (%) | 55 (18.0%)  127 (42.3%)  74 (24.7%)  60 (20.0%)  56 (18.7%)  38 (12.7%)  26 (8.7%)  6 (2.0%) |
| Cardiovascular disease (more than one risk factor or disease per patient possible)  Rhythm abnormality  Coronary artery disease  Previous myocardial infarction  (Minor) stroke  Peripheral artery disease  Heart failure  Left ventricular hypertrophy  Valve abnormality  Aortic aneurysm  Thrombo-embolic event’  Other (congenital heart disease, perimyocarditis, cardiomyopathy, dilated cardiomyopathy, left bundle branch block, sick sinus syndrome, conduction  disorder, pericarditis, recovered cardiomyopathy, ischemic cardiomyopathy) | 43 (14.3%)  38 (12.7%)  23 (7.7%)  23 (7.7%)  14 (4.7%)  12 (4.0%)  8 (2.7%)  8 (2.7%)  7 (2.3%)  6 (2.0%)  10 (3.3%) |
